# Supplementary material for: Exploring the roles of male partners in the transmission, prevention and control of cervical cancer in Central Kenya: A qualitative study
Source: PLoS One. 2025 Sep 11;20(9):e0324575. doi: 10.1371/journal.pone.0324575 (PMC12425210; doi:10.1371/journal.pone.0324575)
Supplement: S1 Table — (PDF) [file pone.0324575.s001.pdf]

Study sites and characteristics of participants for interviews.

Participant Code	Name of the facility	Age (years)	Gender	Years of experience	Level of qualification	Category
N_1	Murang'a County Referral Hospital	34	Female	10	Diploma(KRCHN)	Nurse
N_2	Murang'a County Referral Hospital	33	Male	9	Diploma(KRCHN)	Nurse
N_3	Murang'a County Referral Hospital	36	Female	12	Diploma(KRCHN)	Nurse
N_4	Murang'a County Referral Hospital	28	Female	5	Diploma(KRCHN)	Nurse
N_5	Murang'a County Referral Hospital	44	Female	20	Degree (BSCN)	Nurse
N_6	Murang'a County Referral Hospital	34	Male	9	Diploma(KRCHN)	Nurse
N_7	Murang'a County Referral Hospital	23	Female	1	Diploma(KRCHN)	Nurse
N_8	Nyeri County Referral Hospital	47	Female	23	Diploma(KRCHN)	Nurse
N_9	Nyeri County Referral Hospital	55	Female	32	Diploma(KRCHN)	Nurse
N_{10}	Nyeri County Referral Hospital	38	Female	14	Degree (BSCN)	Nurse
N_{11}	Nyeri County Referral Hospital	40	Female	16	Diploma(KRCHN)	Nurse
N_{12}	Nyeri County Referral Hospital	25	Female	3	Diploma(KRCHN)	Nurse
N_{13}	Nyeri County Referral Hospital	37	Male	12	Diploma(KRCHN)	Nurse
N_{14}	Nyeri County Referral Hospital	40	Female	15	Diploma(KRCHN)	Nurse
N_{15}	Kerugoya County Referral Hospital	45	Female	21	Diploma(KRCHN)	Nurse
N_{16}	Kerugoya County Referral Hospital	36	Female	12	Diploma(KRCHN)	Nurse
N_{17}	Kerugoya County Referral Hospital	27	Female	3	Degree (BSCN)	Nurse
N_{18}	Kerugoya County Referral Hospital	35	Female	9	Diploma(KRCHN)	Nurse

N_{19}	Kerugoya County Referral Hospital	27	Female	3	Diploma(KRCHN)	Nurse
N_{20}	Kerugoya County Referral Hospital	54	Female	30	Diploma(KRCHN)	Nurse
CO_1	Murang'a County Referral Hospital	42	Male	15	Diploma	Clinical Officer
CO_2	Nyeri County Referral Hospital	47	Male	24	Diploma	Clinical Officer
CHW_1	Murang'a County Referral Hospital	48	Female	10	High school	Community Health Worker
CHW_2	Murang'a County Referral Hospital	43	Male	4	High school	Community Health Worker
CHW_3	Nyeri County Referral Hospital	54	Male	15	Primary school	Community Health Worker
CHW_4	Nyeri County Referral Hospital	40	Female	10	High school	Community Health Worker
CHW_5	Kerugoya County Referral Hospital	45	Female	15	High school	Community Health Worker
CHW_6	Kerugoya County Referral Hospital	51	Male	22	Primary school	Community Health Worker
G_1	Murang'a County Referral Hospital	47	Male	19	Masters	Gynecologist
G_2	Nyeri County Referral Hospital	52	Male	22	Masters	Gynecologist
CDH_1	Murang'a County Referral Hospital	49	Male	2	Degree	County Director of Health
CDH_2	Nyeri County Referral Hospital	52	Male	2	Degree	County Director of Health
CDH_3	Kerugoya County Referral Hospital	47	Male	2	Degree	County Director of Health

Demographic characteristics of participants in focus groups discussion

**Participant code**	**Gender**	**Age in years**	**Level of Education**	**Health Facility Name**
$FGD1_{Y1}$	Male	35	Secondary school	Murang'a
$FGD1_{X1}$	Female	30	Secondary school	"
$FGD1_{Y2}$	Male	28	Secondary school	"
$FGD1_{X2}$	Female	24	Secondary school	"
$FGD1_{Y3}$	Male	37	College	"
$FGD1_{X3}$	Female	32	Secondary school	"
$FGD1_{Y4}$	Male	40	Secondary school	"
$FGD1_{X4}$	Female	37	Primary school	"
$FGD1_{Y5}$	Male	50	College	"
$FGD1_{X5}$	Female	43	College	"
$FGD1_{Y6}$	Male	44	Secondary school	"
$FGD1_{X6}$	Female	38	Secondary school	"
$FGD1_{Y7}$	Male	24	Primary school	"
$FGD1_{X7}$	Female	21	Primary school	"
$FGD2_{Y1}$	Male	28	Secondary school	Nyeri
$FGD2_{X1}$	Female	29	Secondary school	"
$FGD2_{Y2}$	Male	34	Secondary school	"
$FGD2_{X2}$	Female	22	Primary school	"
$FGD2_{Y3}$	Male	35	Secondary school	"
$FGD2_{X3}$	Female	30	Secondary school	"
$FGD2_{Y4}$	Male	45	Secondary school	"
$FGD2_{X4}$	Female	38	Secondary school	"
$FGD2_{Y5}$	Male	45	Secondary school	"
$FGD2_{X5}$	Female	37	Secondary school	"
$FGD2_{Y6}$	Male	35	Secondary school	"
$FGD2_{X6}$	Female	28	Secondary school	"
$FGD2_{Y7}$	Male	29	Secondary school	"
$FGD2_{X7}$	Female	23	Secondary school	"
$FGD3_{Y1}$	Male	37	College	Kerugoya
$FGD3_{X1}$	Female	30	College	"
$FGD3_{Y2}$	Male	27	Secondary school	"
$FGD3_{X2}$	Female	22	Primary school	"
$FGD3_{Y3}$	Male	45	Secondary school	"
$FGD3_{X3}$	Female	31	Primary school	"
$FGD3_{Y4}$	Male	38	Secondary school	"
$FGD3_{X4}$	Female	27	Secondary school	"

$FGD3_{Y5}$	Male	46	College	“
$FGD3_{X5}$	Female	41	Secondary school	“
$FGD3_{Y6}$	Male	36	Secondary school	“
$FGD3_{X6}$	Female	27	Secondary school	“
