# Supplementary material for: Exploring the roles of male partners in the transmission, prevention and control of cervical cancer in Central Kenya: A qualitative study
Source: PLoS One. 2025 Sep 11;20(9):e0324575. doi: 10.1371/journal.pone.0324575 (PMC12425210; doi:10.1371/journal.pone.0324575)
Supplement: S3 File — (PDF) [file pone.0324575.s004.pdf]

Annexure P: Focused group interview transcript 1

Study: Developing a logical model for sustainable male partner involvement in cervical cancer prevention and control in Kenya
Participants: Seven Couples attending MCH clinic in Murang'a county referral hospital
Interviewer: John Hiuhu Mwangi
FDG code: 0001
Date: Tuesday 16th April 2024
Duration: 86 minutes and 44 seconds
Place of interview: Murang'a county referral Hospital MCH Nursing staff tea room

Interviewer: So, it *[voice recorder]* has started recording our conversation. As already alluded to earlier, what we are going to discuss among ourselves is the concept of the roles of male partners in cervical cancer transmission, prevention and control. We are also going to discuss the challenges and barriers affecting male partners in participating in cervical cancer prevention and control processes. For today's session I have few questions for you, and under each question there might be probing that may happen and please feel free to voice your thoughts throughout. So, we are not going to talk err… Just feel free to say whatever you want to and say throughout, we don't need to raise up our hands. So, colleagues, the first question is, err do you think male partners should be involved/participate in cervical cancer screening?
[Silence...] anybody can speak.
Participant 1 (Male): eer…I will go first. I think we should be involved only that sometimes circumstances do not allow us

Commented [JM1]: Priority and time constraint

Interviewer: Thank you for your answer sir, you have talked about circumstances, yes...What circumstances? Kindly elaborate.
Participant 1 (Male): you know most of the time we might be busy, for my case am business person and due to my nature of work Am a maize broker and I travel a lot, I usually travel to areas they are harvesting maize as that time the selling price from farmers is low, so if my madam is coming to the clinic I will prioritize business first.

Commented [JM2]: Time constraint

Commented [JM3]: priority

Participant 2 (Female): Yes they should be involved, they are our husbands and they should be there when we are being checked the way they would like us to be there for them in case they are the one being checked.

Commented [JM4]: moral supprt

Interviewer: Mrs. X2. thank you for your answer, actually you have done well to introduce our second question, now that you have said your husbands should be involved, eer...how can they be involved.

Participant 2 (Female): They should ensure we have enough cash to cater for transport and drugs. In case they are available they should accompany us too.

Commented [JM5]: financial

Interviewer: Thank you Mrs. X2 for your answer, I should clarify that they are no drugs that are used during cervical cancer screening process and I agree with you on funds provision. Mrs. X3 we agreed no raising of hands but you can continue…

Participant 3 (female): Sorry about that. Yes, he should take me there for support in case I am told I have cancer. Cancer diagnoses is very scary and one should not be alone in case the diagnoses comes positive.

Commented [JM6]: moral support

Interviewer: Thank you Mrs. X3 for your answer and I need to explain that screening for cervical cancer is not the same as diagnosing the disease. Here we do VIA test which is the use of acetic acid on the cervix to establish whether there are abnormal lesions. In case the test is positive the woman is sent for further tests in the lab.

Participant 1(male): Now that you have mentioned screening**,** What about cervical cancer screening services? Is it free in all public hospitals in Kenya? Some of these services are very expensive, last week we were charged Ksh 3000 for ultra sound for my wife.

Commented [JM7]: financial issues

Commented [JM8]: financial

Interviewer: Thank you Mr. YI for you question, the screening services are free, you only need to pay the 100 for registration which is routine for all the services here. Do you know how cervical cancer is got? …Anyone
Participant 4 (male): I understand all cancers are by chances. You either get or you don't.
Participant 5 male: as for me I think cancers are brought about by our lifestyles including diet and behaviors
Participant 5 (female): I heard somewhere that cervical cancer can be brought about by sleeping with uncircumcised men. (laughter………)
Participant 7 (male): Me I think cancer is curse or bad omen. Either there is a familiar curse that need to be addressed through cleansing ceremonies or traditional sacrifices
Participant 3 (female): Are you saying all the people with cancer are cursed? I think you are being unfair…My Aunt died of breast cancer and she was the best person that I have ever met….
Interviewer: Excuse me guys…we agreed no answer is wrong and everyone is giving his or her opinion. Then here were are focusing on cervical cancer not all cancers.97% of all cervical cancer are caused by Human Papilloma Virus which is a sexually transmitted infection, so what am trying to say is that male partners can actually directly contribute to their women getting cervical cancer.
Participant 6 (male): Cancer is not an infection how can it be transmitted sexually
Participant 5 (female): Do you mean those women with cervical cancer got it from their partners
Interviewer: Thank you for your questions and concerns. It is a fact that cervical cancer can be sexually transmitted and also possible women with the cervical cancer got it from HPV infections from their husbands and that is why it's important for male partners to take an active role in HPV transmission prevention. The good news is that we have vaccine against HPV. How many of you know about HPV Vaccine and have you taken your children for vaccination of the same?
Participant 3 (female): Yes, I know about the vaccine and Mine is vaccinated…two doses, this vaccine is safe…cancer is painful and expensive to treat, kindly let your daughter be vaccinated and she will be safe as mine. "
Participant 6 (male): I don't trust this vaccine they are giving our girls at school; small girls cannot get cervical cancer."

Commented [JM9]: lack of knowledge

Commented [JM10]: lack of knowledge/myths

Commented [JM11]: myths and misconceptions

Commented [JM12]: misconceptions

Commented [JM13]: lack of awareness

Commented [JM14]: lack of awareness

Commented [JM15]: financial issues

Commented [JM16]: lack of knowledge and myths

Interviewer: Thank you Mrs. X5 and Mr. Y6. For your contribution. The vaccine is safe and effective and has been used for more than 10 years in different countries without any major adverse effects.

Participant 8 (female): What age are girls supposed to get the vaccine?

Commented [JM17]: lack of awareness

Interviewer: Thank you Mrs. X 8 for the question. First the vaccine is not for girls only but also for boys, since everyone is at risk of HPV infection. Then the optimal time to get HPV vaccine is before the first sexual debut which is usually between 9 and 13 years**.**
Participant 8 (female): Thank you doctor for your answer.

Interviewer: What characteristics should a health care provider offering cervical cancer prevention and control have to ensure sustainable provision of the services to the males support their partners during the process?
Participant 9 (female): in my opinion they should be courteous and welcoming. Most of them are usually quarrelsome for no apparent reason
Participant 10 (female): I think they should be nonjudgmental and welcoming to us and our wives.

Commented [JM18]: health workers characteristics

Commented [JM19]: health workers characteristics

Commented [JM20]: health workers characteristics

Participant 11 (male): They should be knowledgeable and skilled

Commented [JM21]: health workers characteristics

Participant 12(female): They should be confident and polite

Commented [JM22]: health workers characteristics

Participant 13 (male): There need to be more male health workers as majority are women in these clinics

Commented [JM23]: health care organization

Interviewer: If am getting you right Mr. Y7, you are saying we should have more male nurses, and if so why?

Participant 13 (male): Yes, male nurses or clinical officers. This will encourage us to come as the way things are now it seems as these clinics are meant for women alone.

Commented [JM24]: health care organization

Commented [JM25]: lack of knowledge/awareness

Interviewer: If I may go back, Mrs X5 You said sometimes the nurses are quarrelsome, kindly elaborate**.**

Participant 9 (female): During the delivery of my second born, the nurses in maternity were very rude to me. My husband had accompanied me and I don't think he was encouraged when i reported to him how I was handled.

Commented [JM26]: health workers characteristics

Participant 14 (male): Its true what my wife is saying, those midwives were rude and in case we get another child we won't sought services there

Commented [JM27]: health workers characteristics

Interviewer: Am sorry Mrs. X7 and Y7 on what happened to you. Though am not saying you ere uncooperative, sometimes the nurses are firm during the second stage of labor due to the nature of the labor progress and the fetal wellbeing. None the less, no health worker should be rude to clients or patients.

Participant 2 (female): Some nurses take their children to private clinics—does it mean they don't trust their own services?"

Commented [JM28]: lack of awareness

Interviewer: Thank you Mrs. X1 for your concern, I believe nurses here trust the health services they provide but sometimes some of the services and consumables may not be available in our government facilities, so the staff may source them in other private or non-governmental entities.

Participant 1 (male): When my wife was told that the baby was not positioned well during her pregnancy, the health workers here were very supportive and really helped us until the baby was delivered.

Commented [JM29]: health care characteristics

Interviewer: Thank you Mr. Y1 for your input, atleast there is a positive view, I thought everything is gloomy…(laughter)…It's me believe most of health workers here are professionals How can clinics or health centers be organized so that males accompanying their partners are comfortable to ensure their continued support during cervical cancer prevention and control health service provision of the services?

Participant 4(male): The sitting arrangement is so squeezed—do you expect us to fight for seats with pregnant women and children?"

Commented [JM30]: health care system layout

Participant 6 (male): They should provide more toilets and also clean the available ones regurally. Sometimes we are forced to share washrooms with women here.

Commented [JM31]: health care system lay out

Interviewer: Thank you Mr. Y4 and Y6, I agree with you about the layout and something need to be done if we are to encourage male partners to be coming here. I will talk with the matron about it. Finally, my last question**,** what support should be provided to couples and community to ensure sustained male partner involvement in cervical cancer prevention and control?

Participant 3 (female): I think they need to make all these services free and offer all of them at the same place, the other day they examined me and said I should go for further tests. I didn't have the money, since we had paid fees for our son in college. The way the economy is, majority of us here will not afford these tests. As you explained thy are free here but if they tell you to do more tests, there will be monitory implications. When there cost of services are unaffordable everyone including male partners will be discouraged.

Commented [JM32]: financial

Commented [JM33]: financial

Commented [JM34]: financial

Commented [JM35]: financial

Participant 5 (female:): Personally I think men should be counselled about cervical cancer and other reproductive health issues. Also cervical cancer preventive services including HPV vaccination and testing should be made available to all. The education and counselling can be done at the community level by the community health workers since that is where they will access more male partners

Commented [JM36]: opportunities

Commented [JM37]: opportunities

Interviewer: Thank you Mrs. X5 and X3 for your contribution, they are very important inputs. Is there anyone with a question or comment?

Participant 3 (female): I don't have a question, only to thank you for your time and inviting us for this part of your study, it was more of an educative session than a study and we are very grateful.

Interviewer: Thank you Mrs. X5 and everyone else for your time and accepting to be part of my study. Am really grateful. Make sure you pass to my assistant here to get your transport allowance.*(…recorder stopped*)
