# Supplementary material for: Exploring the roles of male partners in the transmission, prevention and control of cervical cancer in Central Kenya: A qualitative study
Source: PLoS One. 2025 Sep 11;20(9):e0324575. doi: 10.1371/journal.pone.0324575 (PMC12425210; doi:10.1371/journal.pone.0324575)
Supplement: Attachment — Submitted filename: Review Comments.docx [file pone.0324575.s005.docx]

Thank you for the opportunity to review this manuscript. This is a valuable manuscript with meaningful insights into cancer prevention and management in LMICs. However, some sections require clarification, statistical rigor, and better alignment between objectives, results, and conclusions. Improvements in structure, clarity, and depth of interpretation would enhance its contribution to public health literature. Below are specific comments on the manuscript;

**Title**

-Consider improving grammatical agreement and clarity of focus to enhance precision and readability.

-Consider changing **"male partner"** to **"male partners"** to match plural usage unless the study focused on a single partner per participant.

- Replace “A Qualitative Approach” with “A Qualitative Study” for conciseness.

-You can write it this way or otherwise: "Exploring the Roles of Male Partners in the Transmission and Prevention of Cervical Cancer in Central Kenya: A Qualitative Study"

**Abstract**

**Background**

-The phrase “While the it primarily affects women” contains a grammatical error (“the it” is redundant). It should read “While it primarily affects women.”

-The statement “men play a crucial role in the transmission of HPV and also influence the processes of prevention and control” is broad and lacks specificity about how men influence these processes (e.g., behaviors, decision-making).

-The background does not connect to implementation challenges (e.g., barriers to male involvement in prevention programs), which would strengthen its relevance to implementation science.

**Aim**

-The phrase “The study aimed at exploring couples, health care workers and policy makers perceptions” is grammatically incorrect and lacks possessive form or an apostrophe. It should read “couples’, healthcare workers’, and policymakers’ perceptions.”

-The term “male partner” in the aim is singular, inconsistent with the title’s plural “MALE PARTNER” (noting the title’s error). It should be “male partners” for consistency

**Setting**

-The setting is hospital-based, but the study includes community health workers and couples, suggesting data collection may extend beyond hospitals (e.g., community settings). This discrepancy needs clarification.

-Specific counties (e.g., Kiambu, Nyeri) are not named, which would enhance precision, especially for a regional study

**Methods**

-The methods section lacks many key issues like sampling approach, trustworthiness, etc.

-There is no qualitative research design. Please read and reflect on what you did and report accordingly the research approach and design

**-**The participant breakdown is detailed but inconsistent in formatting (e.g., “20 couples,20 Nurses two clinical officers two gynecologists, six Community health workers and three county directors of health”). It lacks punctuation and capitalization consistency (e.g., “Nurses” vs. “nurses,” “Community health workers” vs. “health workers”).

-The total of 73 participants is stated, but it’s unclear how “20 couples” translates to individuals (e.g., 40 individuals if both partners were interviewed). This needs clarification.

-The methods lack mention of data collection methods (e.g., interviews, focus groups), which is critical for understanding the qualitative approach.

**Results**

-Please summarize and report only the key findings

**-**The phrase “Based on the participants perceptions” is grammatically incorrect (missing possessive form). It should read “Based on participants’ perceptions.”

-The results are broad and lack specific examples or depth (e.g., what myths/misconceptions? what cultural practices?). This limits the abstract’s informativeness.

-The term “behavior towards HPV spread” is vague; it could be clarified as “sexual behaviors contributing to HPV transmission.”

**Conclusion**

-Highlight implications more concretely in the conclusion, e.g., specific interventions.

-The phrase “There is lack of awareness” is grammatically awkward and should be “There is a lack of awareness” or “Awareness is limited.”

-The conclusion is brief and does not emphasize implications for practice or policy, which is critical for a public health study.

-The term “males” is inconsistent with “male partners” used elsewhere; “male partners” should be used for consistency.

**Keywords**

-Consider reviewing the keywords to ensure they are all MeSH terms

**Background**

-Streamline the background to ensure that each paragraph is comprehensive and communicates a single idea. For example, paragraph one should only report about the magnitude and burden of cervical cancer from global perspectives to local perspectives, then paragraph two should report what has been done about the problem.

-Contextualize Kenya’s Cervical cancer burden by adding recent national prevalence data

- Streamline the literature review to reduce redundancy, remember to put appropriate intext citation and avoid including outdated references.

-Provide a clear research question or objectives at the end.

-It seems like the references do not match what has been reported in the text.

**Methodology**

-The methodology section is poorly arranged, please revise it entirely and reorganize

-Revise the study design description to clearly articulate the approach and design used with appropriate citation on why you employed such approach and design as far as context of your study is concerned.

-The study setting is not described comprehensively, rather it is stated out. Please revise and explain why you selected that setting and not others

-The sample and sampling procedure are not well articulated. Please describe them in detail, so that the readers can understand well the protocols undertaken for your study.

-Your tables are not cross-referenced. Consider cross-referencing your tables after you have re-analyzed your data

**Results**

-In qualitative study, we prefer to use the term “findings” rather than “results”

-The analysis is poorly performed and there is an overlap between the themes and redundancy of information including quotations. Please reanalyze again your transcript according to thematic analysis as described by Braun and Clarke, <https://www.tandfonline.com/doi/abs/10.1191/1478088706QP063OA?casa_token=0BCmSV7WAq0AAAAA:Q0509jaFWXmyxHe6nR9BqmaP6Yr4-0W757nPcFpNqWgLGNGVS0nK592lpNS_922_s47pP8TT3XSbbA>

-After you have reanalyzed your data, and read to understand how qualitative findings are described and reported.

**Discussion**

-The discussion is more centered on repeating more of the results of the study, which is not acceptable.

-After you have re-analyzed your data, rewrite the discussion section to entail the following aspects;

- Summarize your findings in relation with your study objectives
- Compare and contrast your findings with other findings from diverse researches
- Justify the congruency and incongruency for the comparisons done per each key and significant finding
- Provide an implication (clinical, policy etc.,) for each key and significant finding

-Revise the study limitations to include methodological-related constraints

-Revise the recommendations to reflect what you found please

-The conclusions provided is irrelevant to your findings. Revise the conclusions to stem from your findings and remember that conclusion is not a summary of your study rather an analysis of your study with greater focus on what you found.

**References**

-The references are poorly reported. Why the authors names capitalized?

-Does the citation match the journal format?

-The references seem to be unrelated to the what has been reported and described in the whole manuscript.

-Consider the use of reference managers to cite appropriately and maintain scientific integrity of your work.
