# Supplementary material for: Exploring the roles of male partners in the transmission, prevention and control of cervical cancer in Central Kenya: A qualitative study
Source: PLoS One. 2025 Sep 11;20(9):e0324575. doi: 10.1371/journal.pone.0324575 (PMC12425210; doi:10.1371/journal.pone.0324575)
Supplement: Attachment — Submitted filename: Response to Reviewers (1).docx [file pone.0324575.s008.docx]

8^th^ July 2025,

Editor,

Plos One.

## RE: Response to Review for Manuscript ID: PONE-D-25-21773

## Thank you for your review of our manuscript entitled ***“****Exploring the roles of male partner in transmission and prevention of cervical cancer in Central Kenya: a qualitative approach”*

## and the opportunity to resubmit a revised version.

We appreciate the editor and reviewers’ valuable feedback, and we are pleased to incorporate these recommendations. We are resubmitting the revised manuscript that addresses all concerns. Below is an updated point-by-point response to the comments along with a description of the changes that have been made with reference to the tracked document. The Academic editor and reviewers comments are included in bold text, and our responses are in standard font. We appreciate, in advance, your time and consideration of this revised manuscript for publication in the Plos One journal.

Thank you very much for the opportunity to address these suggestions and concerns and to revise our manuscript. We look forward to a favorable consideration.

Sincerely,

John Hiuhu Mwangi

Corresponding Author.

**Editor Comments
Title and Abstract
1. Revise the title for grammatical accuracy, clarity, and consistency (e.g., use “male partners” and “A Qualitative Study” as suggested)**

We have reviewed the title which now reads. Exploring the Roles of Male Partners in the Transmission, Prevention and control of Cervical Cancer in Central Kenya: A Qualitative Study **2. The abstract requires grammatical corrections, more specificity regarding male partners’ roles, and a clearer connection to barriers in male involvement and implementation challenges**

We have clarified this and reviewed the result section. **3. Ensure consistency in terminology (e.g., “transmission,” “prevention,” and “control”) throughout the manuscript and between the title, abstract, and aims**

This is clarified and is now consistent. **4. Background
• Reorganize the background to provide a logical flow: begin with the global and regional burden of cervical cancer, then contextualize the situation in Kenya and LMICs
• Avoid redundancy and ensure all references are current, relevant, and properly cited
• Clearly state the research question or objectives at the end of the background section**

We have reviewed the background to first contextualize cervical cancer in Kenya; we have also updated all the references and included the research objective in the last paragraph of the introduction.  **5. Methods
• The methodology section requires a complete overhaul for clarity and transparency:
o Clearly describe the qualitative research design, sampling approach, participant selection, and data collection methods (e.g., interviews, focus groups)**

We thank the editor for this important note. We have reviewed the method section for clarity.

**6. Clarify inconsistencies in participant numbers and sampling procedures, especially regarding couples and the use of both purposive and systematic sampling**

We have clarified that only purposive sampling was used in the study. And the 20 couples interviewed (40 participants) clarified. Line 115-117,124-125. **7. Provide detailed information about the study settings, including characteristics of the hospitals and rationale for their selection**

We have clarified this in the study setting section by providing cervical cancer incidence cases as well as cervical cancer services offered in the health facilities. Line 105-110 **8. Explain how data saturation was determined for each participant group**

We have clarified this in the methods section line 121-122,141-143,158-160 **9. Ensure all acronyms are defined upon first use**

We have defined all acronyms at their first mention.  **Results/Findings
10. Rename this section as “Findings” to align with qualitative research conventions**

We have renamed this as ‘Findings’ in line 190 . **11. Reanalyze the data using a recognized qualitative analysis framework (e.g., Braun and Clarke’s thematic analysis) and ensure that themes and subthemes are logically developed and supported by appropriate participant quotes**

We have reviewed the result section and deleted quotes that are repetitive and not reflective of the statements.  **12. Avoid redundancy and overlap between themes; ensure that each theme directly addresses the study objectives. Clarify the distinction between perceived roles and challenges, and adjust themes accordingly.**

We have reviewed the entire result section.

**13. Discussion
• Revise the discussion to:
• Summarize findings in relation to the study objectives
• Compare and contrast findings with existing literature, providing justification for similarities and differences
• Discuss the implications of findings for clinical practice, policy, and future research
• Address methodological limitations more comprehensively**

We have reviewed the entire discussion section as advised. We have also included a section on Implications for Future Research, Practice, and Policy

**14.Conclusion
• Revise the conclusion to ensure it stems directly from the findings, highlighting the implications for practice and policy rather than merely summarizing the study
• Ensure consistency in terminology and alignment with the revised findings**

We have reviewed the conclusion to be reflective of our findings in line 483-492. **15. References
• Review and correct all references for accuracy, relevance, and adherence to journal formatting guidelines
• Use a reference manager to ensure proper citation and scientific integrity**

We have reviewed all the references for accuracy and used a reference manager. **9. Language and Formatting
• Thoroughly proofread the manuscript to correct typographical, grammatical, and formatting errors
• Ensure consistent use of terms and proper structure throughout the manuscript**

We thank the editor for this important note. We have reviewed the manuscript for typographical, formatting and grammar errors.

**Reviewer 1**

Thank you for the opportunity to review this manuscript. This is a valuable manuscript with meaningful insights into cancer prevention and management in LMICs. However, some sections require clarification, statistical rigor, and better alignment between objectives, results, and conclusions. Improvements in structure, clarity, and depth of interpretation would enhance its contribution to public health literature. Below are specific comments on the manuscript;

**Title**

1. **Consider improving grammatical agreement and clarity of focus to enhance precision and readability. Consider changing "male partner" to "male partners" to match plural usage unless the study focused on a single partner per participant.**

We have made this change in the title now reads male partners.

2. **Replace “A Qualitative Approach” with “A Qualitative Study” for conciseness.**

We have made this change.

3.**You can write it this way or otherwise: "****Exploring the Roles of Male Partners in the Transmission and Prevention of Cervical Cancer in Central Kenya: A Qualitative Study"**

Thank you for this important note. We have adopted this change in the title.

**Abstract**

**4. Background: The phrase “While the it primarily affects women” contains a grammatical error (“the it” is redundant). It should read “While it primarily affects women.”**

We have made this change in the abstract section line 9.

**5. The statement “men play a crucial role in the transmission of HPV and also influence the processes of prevention and control” is broad and lacks specificity about how men influence these processes (e.g., behaviors, decision-making).**

We have made this change in the background line 8-12

**6. The background does not connect to implementation challenges (e.g., barriers to male involvement in prevention programs), which would strengthen its relevance to implementation science.**

We have revised the background and line 8-12 now reads. Cervical cancer (CC), primarily caused by persistent infection with high-risk human papillomavirus (HPV) types, remains a major global public health issue. While it primarily affects women, male partners significantly influence HPV transmission, as well as women's access to prevention and treatment through decision-making and social support. However, their role in treatment engagement remains underexplored.

**7. Aim. The phrase “The study aimed at exploring couples, health care workers and policy makers perceptions” is grammatically incorrect and lacks possessive form or an apostrophe. It should read “couples’, healthcare workers’, and policymakers’ perceptions.”**

We have made this change in line 13-15 the aim now read. “The study aimed to explore the perceptions of couples’, healthcare workers’, and policymakers’ regarding the role of male partners in the transmission, prevention, and control of cervical cancer.”

**8. The term “male partner” in the aim is singular, inconsistent with the title’s plural “MALE PARTNER” (noting the title’s error). It should be “male partners” for consistency**

This have corrected and now reads ‘male partners’ in the entire document.

**9. Setting: The setting is hospital-based, but the study includes community health workers and couples, suggesting data collection may extend beyond hospitals (e.g., community settings). This discrepancy needs clarification.**

We have clarified this which now reads ‘The study was carried out in three public county hospitals and community settings in Nyeri, Murang’a and Kirinyaga counties in Central Kenya’. line 16-17.

**10. Specific counties (e.g., Kiambu, Nyeri) are not named, which would enhance precision, especially for a regional study**

This is clarified in line 17 which now reads ‘Nyeri, Murang’a and Kirinyaga counties in central Kenya.’ Kiambu county was not sampled.

**11. Methods: The methods section lacks many key issues like sampling approach, trustworthiness, etc. There is no qualitative research design. Please read and reflect on what you did and report accordingly the research approach and design**

This has been rectified. Line 18-23

**12. The participant breakdown is detailed but inconsistent in formatting (e.g., “20 couples,20 Nurses two clinical officers two gynecologists, six Community health workers and three county directors of health”). It lacks punctuation and capitalization consistency (e.g., “Nurses” vs. “nurses,” “Community health workers” vs. “health workers”).**

We have reviewed this inconsistency line 18-23 now reads “We used qualitative research approaches for a comprehensive exploration of the roles of male partners in CC transmission, prevention and control. We conducted in-depth interviews and focus group discussions with 73 participants including; 20 couples, 20 Nurses two Clinical officers 2 Gynecologists, 6 Community health workers and 3 County directors of health” The couples were considered two individuals participants as they answered questions independently.

13.**Your tables are not cross-referenced. Consider cross-referencing your tables after you have re-analyzed your data.**

We have cross referenced the tables**.**

**13. The total of 73 participants is stated, but it’s unclear how “20 couples” translates to individuals (e.g., 40 individuals if both partners were interviewed). This needs clarification.**

We have clarified this in the findings section. Line 192 reads “Overall, 20 couples (n=40) participants participated in the focused group discussion”. A couple was considered as two participants as they both answered some questions independently.

**14. The methods lack mention of data collection methods (e.g., interviews, focus groups), which is critical for understanding the qualitative approach.**

We have clarified this in line 18-23.

**15. Results: Please summarize and report only the key finding**

We have reviewed the result section to reflect the key findings. Line 24-33.

**16. The phrase “Based on the participants perceptions” is grammatically incorrect (missing possessive form). It should read “Based on participants’ perceptions**.”

We have rectified this by redoing the whole part.

17. **The results are broad and lack specific examples or depth (e.g., what myths/misconceptions? what cultural practices?). This limits the abstract’s in formativeness.**

We have rectified this by redoing the whole section of results in the abstract. Line 24-33

**18. The term “behavior towards HPV spread” is vague; it could be clarified as “sexual behaviors contributing to HPV transmission.”**

The whole part of the results has been reorganized. Line 24-33.

**19. Conclusion: Highlight implications more concretely in the conclusion, e.g., specific interventions.**

The conclusion is reviewed. Line 34-36 now reads “Cervical cancer awareness is limited, and male support is shaped by financial, cultural, social, and health system factors. Addressing these issues is key to improving prevention and control efforts.

20. **The phrase “There is lack of awareness” is grammatically awkward and should be “There is a lack of awareness” or “Awareness is limited.”**

We have reviewed this in line 34 now reads “cervical cancer awareness is limited’

21. **The conclusion is brief and does not emphasize implications for practice or policy, which is critical for a public health study.**

We have reviewed the conclusion which ow reads. “Cervical cancer awareness is limited, and male support is shaped by financial, cultural, social, and health system factors. Addressing these issues is key to improving prevention and control efforts”.

22. **The term “males” is inconsistent with “male partners” used elsewhere; “male partners” should be used for consistency.**

We have made this change for consistency.

**23. Keywords: Consider reviewing the keywords to ensure they are all MeSH terms**

Key words now read. Male partner involvement, Cervical cancer, disease transmission and prevention. Line 39.

**24. Background: Streamline the background to ensure that each paragraph is comprehensive and communicates a single idea. For example, paragraph one should only report about the magnitude and burden of cervical cancer from global perspectives to local perspectives, then paragraph two should report what has been done about the problem.**

We have made this change in the first paragraph to highlight global and local perspectives of cervical cancer. The first paragraph now reads. “Cervical cancer (CC) is the fourth most common cause of cancer incidence and mortality among women globally [1]. CC is the most common cause of cancer and cancer deaths among women in Kenya, and 90% of CC deaths occur in low- and middle- income countries (LMIC) [2]. Persistent (long‑lasting) infection with high‐risk human papillomavirus (HPV) types is the necessary cause of virtually all cervical cancers; notably, HPV 16 and 18 together account for approximately 70% of cases worldwide [4]. The 2018 cervical cancer screening guidelines propose to have every woman age 25-49 screened regularly for cervical cancer [5]. Understanding social influences on CC screening and treatment is crucial for better access to medical care.

25.**Contextualize Kenya’s Cervical cancer burden by adding recent national prevalence data.**

We have included the Kenya data in line 44-46. Now reads. “In Kenya, CC is the second most common female cancer and the leading cause of cancer-related death among women aged (15–44), in 2018 there were 3,286 cancer deaths out of the 5250 reported new cases”

26. **Streamline the literature review to reduce redundancy, remember to put appropriate in text citation and avoid including outdated references.**

We thank the reviewer for this important note. We have reviewed the entire introduction section and included only appropriate citations.

27. **Provide a clear research question or objectives at the end.**

We have clarified the research aim at the end of the background section and line 78-82 now reads. “This qualitative study aimed at exploring the roles of male partners in transmission, prevention and control of cervical cancer, as perceived by couples, health care workers and policy makers. This evidence is useful in understanding barriers and facilitators to male partners participation in prevention and treatment of cervical cancer ,which could help inform CC prevention and control programs. ”

**28. It seems like the references do not match what has been reported in the text.**

We have reviewed all the references and used a referencing tool as advised.

**29. Methodology: The methodology section is poorly arranged, please revise it entirely and reorganize**

We thank the reviewer for this important note we have reorganized the entire methods section.

**30.** **Revise the study design description to clearly articulate the approach and design used with appropriate citation on why you employed such approach and design as far as context of your study is concerned.**

We have clarified the study design in lines 86-103.

**31**. **The study setting is not described comprehensively, rather it is stated out. Please revise and explain why you selected that setting and not others.**

We have reviewed the study setting in line 105-110 now reads. “This study was conducted at three county referral hospitals with Maternal and child health (MCH) clinics in Central Kenya: Murang’a, Nyeri, and Kirinyaga. The three public health facilities were purposively selected driven by previously reported high prevalence and incidence of cervical cancer [23]. Additionally, these facilities have recorded more than 2,328 cervical cancer cases between 2017-2020(24). All the three facilities offer cervical cancer screening, cryotherapy treatment and HPV vaccination campaigns(25).”

**32. The sample and sampling procedure are not well articulated. Please describe them in detail, so that the readers can understand well the protocols undertaken for your study.**

We have rectified this in line 112-126. Which now reads. “We conducted in-depth interviews and focus group discussions with 73 participants. These included 20 couples (total of 40 participants) of reproductive age (18- 50 years), health care workers who included 20 nurses, 2 clinical officers, two gynecologists, six community health workers and three county directors of health. Purposive sampling was used to select a diverse range of participants who could offer different perspectives and experiences and who were comfortable providing the information needed for the study. Couples were eligible for the interviews if they were of reproductive age and willing to provide consent, health care workers were eligible if they had prior training on cervical cancer screening and treatment and had worked in the clinics for at least six months, community health workers were stationed within local community, gynecologists and county health directors were deployed in the local counties. Our study sample size was guided by information saturation as in the existing literature (26)

Couples were recruited at the MCH clinics after health talks conducted by the study teams. Purposive sampling was used to select couples to participate in the study based on the eligibility criteria and willingness to participate in the study. The recruitment of the various study participants and data collection process took nine weeks in total from 8^th^ April 2024 to 7^th^ June 2024”.

**34. Results: In qualitative study, we prefer to use the term “findings” rather than “results”**

We have made this change to findings in line 190.

35. **The analysis is poorly performed and there is an overlap between the themes and redundancy of information including quotations. Please reanalyze again your transcript according to thematic analysis as described by Braun and Clarke,** [**https://www.tandfonline.com/doi/abs/10.1191/1478088706QP063OA?casa_token=0BCmSV7WAq0AAAAA:Q0509jaFWXmyxHe6nR9BqmaP6Yr4-0W757nPcFpNqWgLGNGVS0nK592lpNS_922_s47pP8TT3XSbbA**](https://www.tandfonline.com/doi/abs/10.1191/1478088706QP063OA?casa_token=0BCmSV7WAq0AAAAA:Q0509jaFWXmyxHe6nR9BqmaP6Yr4-0W757nPcFpNqWgLGNGVS0nK592lpNS_922_s47pP8TT3XSbbA)

We thank the reviewer for this important note we have rewritten our summaries for clarity and deleted redundant quotations and data.

36. **After you have reanalyzed your data, and read to understand how qualitative findings are described and reported.**

We have made this change in the result section.

**37. Discussion. The discussion is more centered on repeating more of the results of the study, which is not acceptable**.

-**After you have re-analyzed your data, rewrite the discussion section to entail the following aspects;**

- **Summarize your findings in relation with your study objectives**
- **Compare and contrast your findings with other findings from diverse researches**
- **Justify the congruency and in congruency for the comparisons done per each key and significant finding**
- **Provide an implication (clinical, policy etc.,) for each key and significant finding**

We thank the reviewer for this important guidance. We have reviewed the discussion section as advised.

**38. Revise the study limitations to include methodological-related constraints**

We have revised the limitations in line 475-481. It now reads. “This qualitative study provides important findings on male involvement in cervical cancer screening and treatment. However, it is not without limitations. A key limitation is that understanding male partner roles was hypothetical and perceptions may differ with real life experiences with women accessing cervical cancer screening or treatment services. Additionally, participant recruitment was biased among those seeking health care services. As a result, findings may not be generalized to all settings, particularly other resource-limited contexts where health-seeking behaviors and social dynamics may differ.”

**39. Revise the recommendations to reflect what you found please**

We have revised the recommendation to include implication of the findings in research practice and policy. Line 465-473 now reads. “Male partner roles described in our study have significant implications in research practice and policy aimed at strengthening cervical cancer prevention and treatment. First, future research may need to explore male partner roles among women accessing cervical cancer screening and treatment to understand actual involvement. Within health care facilities in practice, health care providers should be trained to actively involve male partners in education and care processes. Education should also address existing myths and cultural beliefs to further strengthen male involvement in cervical cancer care. Within the policy level, male partner engagement is crucial and should be incorporated in national cervical cancer campaigns and mobilization to promote shared responsibility in reproductive health”.

**40.** **The conclusions provided is irrelevant to your findings. Revise the conclusions to stem from your findings and remember that conclusion is not a summary of your study rather an analysis of your study with greater focus on what you found.**

We have revised the conclusions to be based on our findings in line 483-492.It now reads “In conclusion, this qualitative study highlights diverse critical roles male partners play in cervical cancer prevention and treatment, encompassing financial, logistical, emotional, and moral support that significantly alleviate the burden on women and improve treatment adherence. However, knowledge gaps and traditional cultural beliefs create significant barriers to male involvement. Key knowledge gaps should be addressed through targeted education and community engagement is essential to strengthen male partner involvement and enhance the effectiveness of cervical cancer prevention and care. Interventions aimed at increasing male partner engagement, fostering supportive communication, and reducing stigma are likely to have a significant impact on improving cervical cancer care uptake and adherence”.

**41. References**

-**The references are poorly reported. Why the authors names capitalized?**

**-Does the citation match the journal format?**

**-The references seem to be unrelated to the what has been reported and described in the whole manuscript.**

**-Consider the use of reference managers to cite appropriately and maintain scientific integrity of your work.**

We thank the reviewer for this important note. We have revised the citations to reflect what is described in the manuscript. We have also used a reference manager for the manuscript and aligned it with the journal requirements.

**Reviewer 2**

**Reviewer Comments on the study titled “EXPLORING THE ROLES OF MALE PARTNER IN TRANSMISSION AND PREVENTION OF CERVICAL CANCER IN CENTRAL KENYA: A QUALITATIVE APPROACH”**

**Thank you for the opportunity to review this manuscript**

1. **General comments**

- **The manuscript addresses an important area in addressing cervical cancer**
- **There are a number of typographical errors that need to be corrected**
- **The manuscript needs language editing for clarity**
- **Sometime the authors use the terms ‘transmission’ and ‘prevention’ while some other time they include ‘control’. There is a need for being consistent**

We thank the reviewer for these insightful comments. We have done language editing and addressed the typographical errors for clarity. We have also used words transmission, prevention and control consistently.

2. **Abstract: The aim includes the ‘control’ of the condition while this aspect does not feature in the title**

We have made this edit in the title and abstract section for clarity. The study aim now reads “The study aimed to explore the perceptions of couples, healthcare workers, and policymakers regarding the role of male partners in the transmission, prevention, and control of cervical cancer.”

3. **Background: There is a need an overview of cervical cancer and provide the global and regional burden and its context in low- and middle-income countries (LMICs), particularly Sub-Saharan Africa where Kenya is located.**

We have included the overview of cervical cancer in the first paragraph of the manuscript line 42-51.

4. **The structure of this section needs to be revised. Authors present aims in the first paragraph while this should appear at the end of the background.**

We have made this change, and the study aim now appear in the las paragraph of our introduction line 78-82.

5. **Paragraphs need to be presented in a more organized manner. For instance, the first paragraph is loaded with various concepts such as the aims of the study, factors influencing men’s role, and access to sexual education. Transitions from one paragraph to another need to be looked at to improve flow of ideas**

The introduction section is entirely reviewed for flow of idea.

6. **Research Methods and Design: Research design is part of the research methods. The title for this section may need to be revised**

This has been rectified and now reads “Methods” Line 84.

7. **In line 145 MCH is used as an acronym without including its long form at first mention**

We have written this in full for the first mention in line 105 “Maternal and child health (MCH)”

8. **More information is needed to describe the setting of the study especially the characteristics of the three hospitals included (e.g. number of cervical patients served and nature of cervical cancer services provided)**

We have provided additional details in the study setting line 105-110.

9. **It is not clear in line 163 why the authors used simple systematic random sampling to select couples while they have said in line 154 that purposive sampling was employed to select a diverse range of participants who could offer different perspectives. Moreover, it also not clear if all women came to the clinic with their partners (which is not a common practice in many African countries)**

We have clarified the sampling technique in line 123-125. Which now reads. “Couples were recruited at the MCH clinics after health talks conducted by the study teams. Purposive sampling was used to select couples to participate in the study based on the eligibility criteria and willingness to participate in the study”. The researcher had asked the clinic in charges to ask women to come with their partners during their previous visits for this exercise.

10. **In line 190 authors state “The interviews were continued until data saturation was achieved”. However, is not clear exactly how this was done to end up with 20 couples, 20 nurses, 2 clinical officers, 2 gynecologists, 4 community health workers, and 3 county directors of health. Were the questions the same for each group? Was saturation reached for each group or collectively? How saturation was reached for all participants in the three hospitals needs to be clarified.**

We thank the reviewer for this concern. The health care workers including the community health workers were purposefully selected based on their specialty, experience and availability as inclusion criteria (Line 117-121). There is only one County Health director per county so all the three were selected. Information saturation is clarified in the methods section line 121-122,141-143,158-160.

11. **How data was collected for each of the groups of participants needs to be clarifies. For instance, where were in the interviews conducted with community health workers and county directors.**

This is clarified in line 134-137 which now reads “Interviews were conducted at convenient times for the participants and the research assistants. The health professionals and community health workers were interviewed at their work stations while the county health directors were interviewed in their offices”

12. **In line 193 authors state that 3 FGDS were conducted. But the number of participants in each FGDs are not provided for the 40 participants. Moreover, it is not clear whether FGDs were composed of both males and females or not, and why.**

This has been clarified in line 144-147 which now reads” Three FGDs were conducted involving couples from the selected three counties. The distribution of the couples were as follows Nyeri and Murang’a seven couples each (28 participants), Kirinyaga six couples (12 participants) The total number of participants were 40 (20 males and 20 females)

13. **Results. This section should be preferably named as ‘Findings’ which is more relevant to qualitative research**

We have changed ‘Results’ to ‘Findings’ Line 190.

14. **Authors need to ensure the quotes selected describe and support the subthemes and descriptors they have provided. For example, the quote for Subtheme 1.1 (Provision of financial support) in line 286 does not align with males providing financial support but it rather talks about women complaining for lack of funds and free services for screening. Authors should go through all subthemes and ensure the right and rich quotes are used since the presentation of this section seems to consistently have this discrepancy.**

We thank the reviewer for this important note. We have reviewed the quotes to include only relevant quotes. To address this, we have deleted the quote.

15. **Most of the quotes provided show that the findings are more about perceived challenges with males’ involvement in their partners’ cervical cancer issues rather than the perceived roles of males. If this is the case, authors may need to check on this or re-analyze the data and come up with appropriate themes and subthemes.**

We thank the reviewer for this important note. We have reviewed the result section to reflect roles of male partners.

16. **One may wonder how theme no. 4 (Traditional and Cultural practices) with its subthemes (Gender roles and norms and Beliefs and practices) are perceived roles. This shows there was inconsistency in developing themes**

The theme traditional and cultural practices emerged as what influences roles played by men in prevention and treatment. To clarify this, we have reworded it as “Cultural norms Influencing male partner roles”.

17. **In Subtheme 5.2 (Healthcare provision), is not clear how/why healthcare providers are considered part of the roles of male partner in transmission and prevention of cervical cancer.**

Participants reported that male health care providers should be more involved in cervical cancer treating and prevention. Highlighting the role, they would play in offering services such as screening, treatment and referral. We have revised 5.2 for clarity.

18. **Discussion: This section may need revision based on the comments in the findings section**

We have reviewed the entire discussion section.

19. **The section needs also to discuss the implication of findings and not just comparing findings with other studies, as it appears in some parts.**

We have revised the whole of discussion section.

20. **Conclusion: This conclusion primarily emphasizes the challenges of male involvement in their partners’ cervical cancer issues, rather than the perceived roles, as highlighted in the findings section. It may need to be revised to align with any changes made to the findings, ensuring they accurately reflect the study results.**

We have reviewed the conclusion section for clarity.
