# Supplementary material for: Exploring the roles of male partners in the transmission, prevention and control of cervical cancer in Central Kenya: A qualitative study
Source: PLoS One. 2025 Sep 11;20(9):e0324575. doi: 10.1371/journal.pone.0324575 (PMC12425210; doi:10.1371/journal.pone.0324575)
Supplement: Attachment — Submitted filename: Response to reviewers letter.docx [file pone.0324575.s009.docx]

11^th^ August 2025,

Editor,

PLOS ONE.

## RE: Response to Review for manuscript ID: [PONE-D-25-21773R1]

## Thank you for your review of our manuscript entitled ***“****Exploring the Roles of Male Partners in the Transmission and Prevention of Cervical Cancer in Central Kenya: A Qualitative Study****”*** and the opportunity to resubmit a revised version.

We appreciate the editors’ valuable feedback, and we are pleased to incorporate these recommendations. We appreciate, in advance, your time and consideration of this revised manuscript for publication in the Plos One journal.

We have now reviewed the document to verify and adjust references and citations as advised. We have also made changes to grammatical errors in the entire document.

Sincerely,

John Hiuhu Mwangi.

Corresponding Author.
