# Supplementary material for: Exploring the roles of male partners in the transmission, prevention and control of cervical cancer in Central Kenya: A qualitative study
Source: PLoS One. 2025 Sep 11;20(9):e0324575. doi: 10.1371/journal.pone.0324575 (PMC12425210; doi:10.1371/journal.pone.0324575)
Supplement: Attachment — Submitted filename: Reviewer Comments.docx [file pone.0324575.s006.docx]

Reviewer Comments on the study titled “EXPLORING THE ROLES OF MALE PARTNER IN TRANSMISSION AND PREVENTION OF CERVICAL CANCER IN CENTRAL KENYA: A QUALITATIVE APPROACH”

Thank you for the opportunity to review this manuscript

**General comments**

1. The manuscript addresses an important area in addressing cervical cancer
2. There are a number of typographical errors that need to be corrected
3. The manuscript needs language editing for clarity
4. Sometime the authors use the terms ‘transmission’ and ‘prevention’ while some other time they include ‘control’. There is a need for being consistent

**Abstract**

The aim includes the ‘control’ of the condition while this aspect does not feature in the title

**Background**

1. There is a need an overview of cervical cancer and provide the global and regional burden and its context in low- and middle-income countries (LMICs), particularly Sub-Saharan Africa where Kenya is located.
2. The structure of this section needs to be revised. Authors present aims in the first paragraph while this should appear at the end of the background.
3. Paragraphs need to be presented in a more organized manner. For instance, the first paragraph is loaded with various concepts such as the aims of the study, factors influencing men’s role, and access to sexual education. Transitions from one paragraph to another need to be looked at to improve flow of ideas

**Research Methods and Design**

1. Research design is part of the research methods. The title for this section may need to be revised
2. In line 145 MCH is used as an acronym without including its long form at first mention
3. More information is needed to describe the setting of the study especially the characteristics of the three hospitals included (e.g. number of cervical patients served and nature of cervical cancer services provided)
4. It is not clear in line 163 why the authors used simple systematic random sampling to select couples while they have said in line 154 that purposive sampling was employed to select a diverse range of participants who could offer different perspectives. Moreover, it also not clear if all women came to the clinic with their partners (which is not a common practice in many African countries)
5. In line 190 authors state “The interviews were continued until data saturation was achieved”. However, is not clear exactly how this was done to end up with 20 couples, 20 nurses, 2 clinical officers, 2 gynecologists, 4 community health workers, and 3 county directors of health. Were the questions the same for each group? Was saturation reached for each group or collectively? How saturation was reached for all participants in the three hospitals needs to be clarified
6. How data was collected for each of the groups of participants needs to be clarifies. For instance, where were in the interviews conducted with community health workers and county directors
7. In line 193 authors state that 3 FGDS were conducted. But the number of participants in each FGDs are not provided for the 40 participants. Moreover, it is not clear whether FGDs were composed of both males and females or not, and why.

**Results**

1. This section should be preferably named as ‘Findings’ which is more relevant to qualitative research
2. Authors need to ensure the quotes selected describe and support the subthemes and descriptors they have provided. For example, the quote for Subtheme 1.1 (Provision of financial support) in line 286 does not align with males providing financial support but it rather talks about women complaining for lack of funds and free services for screening. Authors should go through all subthemes and ensure the right and rich quotes are used since the presentation of this section seems to consistently have this discrepancy.
3. Most of the quotes provided show that the findings are more about perceived challenges with males’ involvement in their partners’ cervical cancer issues rather than the perceived roles of males. If this is the case, authors may need to check on this or re-analyze the data and come up with appropriate themes and subthemes.
4. One may wonder how theme no. 4 (Traditional and Cultural practices) with its subthemes (Gender roles and norms and Beliefs and practices) are perceived roles. This shows there was inconsistency in developing themes
5. In Subtheme 5.2 (Healthcare provision), is not clear how/why healthcare providers are considered part of the roles of male partner in transmission and prevention of cervical cancer.

**Discussion**

1. This section may need revision based on the comments in the findings section
2. The section needs also to discuss implication of findings and not just comparing findings with other studies, as it appears in some parts

**Conclusion**

This conclusion primarily emphasizes the challenges of male involvement in their partners’ cervical cancer issues, rather than the perceived roles, as highlighted in the findings section. It may need to be revised to align with any changes made to the findings, ensuring it accurately reflects the study results.
